# The Annual Meeting of the Illinois State Medical Society

**Published:** 1886-06

**Authors:** 


### THE ANNUAL MEETING OF THE ILLINOIS STATE MEDICAL SOCIETY

Was held in Bloomington on May 18th and 19th. Dr. W. A. Byrd presided. The attendance of members was scarcely as large as usual, a fact owing doubtless, in part, to the recent meeting of the American Medical Association in St. Louis.

Most of the committees made their reports promptly, and a fair amount of work was done.

The following is a list of the officers elected for the year.

*President*—W. T. Kirk, Atlanta.

*First Vice-President*—E. Wenger, Gilman.

*Second Vice-President*—W. T. Ensign, Rutland.

*Permanent Secretary*—D. W. Graham, Chicago.

*Treasurer*—Walter Hay, Chicago.

*Assistant Secretary*—Henry J. Reynolds, Chicago.

Chicago is the next place of meeting.

The annual meeting will be held in accordance with the constitution, on the third Tuesday in May.

*Committee on Arrangements*—E. Ingals, Etheridge, Starkweather, Foster and Tilley, all of Chicago.

### STANDING COMMITTEES.

*Practice of Medicine*—D. R. Brower, Chicago; A. K. Van Horn, Jerseyville; P. H. Oyler, Mt. Pulaski.

*Surgery*—Dr. D. A. K. Steele, Chicago; C. Goodbrake, Clinton; B. F. Crummer, Warren.

*Obstetrics*—E. A. Ingersoll, Canton; C. DuHadway, Jerseyville; W. H. Conibear, Morton.

*Gynæcology*—O. B. Will, Peoria; J. M. Armstrong, Edwardsville; Catherine Miller, Lincoln.

*Drugs and Medicines*—J. G. Topper, Elgin; T. M. Cullimore, Jacksonville; M. J. Mergler, Chicago.

*Ophthalmology and Otology*—S. J. Jones, Chicago; A. E. Prince, Jacksonville; C. R. Parke, Bloomington.

*Necrology*—E. Ingals, Chicago; William Hill, Bloomington; G. W. Cox, Clayton.

*Publication*—J. F. Todd, Chicago.

*Diseases of Throat and Nose*—E. F. Ingals, Chicago.

*Dermatology*—Henry J. Reynolds, Chicago.

*Diseases of Children*—G. W. Jones, Danville; E. J. Shipp, Petersburg.

*Physiology*—A. Wetmore, Waterloo.

*Judicial Committee*—C. C. Hunt, Dixon; A. T. Barnes; Bloomington; L. G. Thompson, Lacon.

*Medical and Sanitary Legislation*—B. M. Griffith, Springfield; J. L. White, Bloomington; W. A. Haskell, Alton; A. B. Strong, Chicago.

*Biographical*—J. H. Hollister, Chicago; E. P. Cook, Mendota; E. Ingals, Chicago; G. W. Jones, Danville; F. B. Haller, Vandalia; T. F. Worrell, Bloomington; Robert Boal, Peoria; H. R. Guthrie, of Sparta.

*The Incurable Insane*—A. T. Barnes, Bloomington.

---

